# Supplementary material for: Heparin-based hydrogel scaffolding alters the transcriptomic profile and increases the chemoresistance of MDA-MB-231 triple-negative breast cancer cells
Source: Biomater Sci. 2020 Feb 13;8(10):2786–96. doi: 10.1039/c9bm01481k (PMC7497406; doi:10.1039/c9bm01481k)
Supplement: Supplementary file 2 [file BM-008-C9BM01481K-s002.zip › Supplementary File 4/HP-BhydrogelvControl/Pathways/my_analysis.Gsea.1545201079907/HALLMARK_TGF_BETA_SIGNALING.html]

Details for gene set HALLMARK\_TGF\_BETA\_SIGNALING[GSEA]

|  || Dataset | expr.class.cls#HYD\_versus\_CONTROL.class.cls#HYD\_versus\_CONTROL\_repos |
| Phenotype | class.cls#HYD\_versus\_CONTROL\_repos |
| Upregulated in class | HYD |
| GeneSet | HALLMARK\_TGF\_BETA\_SIGNALING |
| Enrichment Score (ES) | 0.27511534 |
| Normalized Enrichment Score (NES) | 0.9290974 |
| Nominal p-value | 0.5813253 |
| FDR q-value | 0.91906214 |
| FWER p-Value | 1.0 |
Table: GSEA Results Summary

  

Fig 1: Enrichment plot: HALLMARK\_TGF\_BETA\_SIGNALING      
 Profile of the Running ES Score & Positions of GeneSet Members on the Rank Ordered List

  

| PROBE | DESCRIPTION (from dataset) | GENE SYMBOL | GENE\_TITLE | RANK IN GENE LIST | RANK METRIC SCORE | RUNNING ES | CORE ENRICHMENT || 1 | SMAD6 | na |  |  | 426 | 2.438 | 0.0393 | Yes |
| 2 | SKI | na |  |  | 584 | 2.235 | 0.0876 | Yes |
| 3 | ID2 | na |  |  | 1383 | 1.637 | 0.0872 | Yes |
| 4 | SMAD7 | na |  |  | 1487 | 1.581 | 0.1217 | Yes |
| 5 | SMURF1 | na |  |  | 1549 | 1.552 | 0.1577 | Yes |
| 6 | PPP1R15A | na |  |  | 1561 | 1.546 | 0.1962 | Yes |
| 7 | NCOR2 | na |  |  | 1743 | 1.474 | 0.2240 | Yes |
| 8 | ENG | na |  |  | 2136 | 1.298 | 0.2363 | Yes |
| 9 | BCAR3 | na |  |  | 2209 | 1.272 | 0.2646 | Yes |
| 10 | SMAD3 | na |  |  | 2896 | 1.064 | 0.2557 | Yes |
| 11 | SMAD1 | na |  |  | 3024 | 1.032 | 0.2751 | Yes |
| 12 | FURIN | na |  |  | 3731 | 0.863 | 0.2600 | No |
| 13 | PMEPA1 | na |  |  | 4320 | 0.748 | 0.2482 | No |
| 14 | NOG | na |  |  | 4562 | 0.698 | 0.2532 | No |
| 15 | HIPK2 | na |  |  | 4827 | 0.654 | 0.2560 | No |
| 16 | BMP2 | na |  |  | 4891 | 0.643 | 0.2689 | No |
| 17 | CDH1 | na |  |  | 5848 | 0.507 | 0.2318 | No |
| 18 | LTBP2 | na |  |  | 5970 | 0.490 | 0.2379 | No |
| 19 | ID3 | na |  |  | 6019 | 0.482 | 0.2475 | No |
| 20 | SMURF2 | na |  |  | 6067 | 0.479 | 0.2571 | No |
| 21 | CDKN1C | na |  |  | 7826 | 0.269 | 0.1721 | No |
| 22 | CDK9 | na |  |  | 8284 | 0.221 | 0.1538 | No |
| 23 | ARID4B | na |  |  | 8324 | 0.216 | 0.1573 | No |
| 24 | FNTA | na |  |  | 9009 | 0.153 | 0.1254 | No |
| 25 | BMPR1A | na |  |  | 9127 | 0.137 | 0.1228 | No |
| 26 | ACVR1 | na |  |  | 9630 | 0.084 | 0.0987 | No |
| 27 | TGFB1 | na |  |  | 9937 | 0.048 | 0.0839 | No |
| 28 | TRIM33 | na |  |  | 10026 | 0.037 | 0.0802 | No |
| 29 | SLC20A1 | na |  |  | 10603 | -0.017 | 0.0506 | No |
| 30 | TGIF1 | na |  |  | 10654 | -0.024 | 0.0486 | No |
| 31 | JUNB | na |  |  | 11042 | -0.071 | 0.0302 | No |
| 32 | KLF10 | na |  |  | 11366 | -0.099 | 0.0158 | No |
| 33 | ID1 | na |  |  | 13064 | -0.293 | -0.0654 | No |
| 34 | APC | na |  |  | 14635 | -0.512 | -0.1345 | No |
| 35 | PPP1CA | na |  |  | 15007 | -0.563 | -0.1396 | No |
| 36 | PPM1A | na |  |  | 15056 | -0.571 | -0.1277 | No |
| 37 | TJP1 | na |  |  | 15122 | -0.585 | -0.1164 | No |
| 38 | HDAC1 | na |  |  | 15229 | -0.601 | -0.1067 | No |
| 39 | MAP3K7 | na |  |  | 15614 | -0.671 | -0.1098 | No |
| 40 | IFNGR2 | na |  |  | 15618 | -0.672 | -0.0930 | No |
| 41 | SKIL | na |  |  | 16151 | -0.777 | -0.1012 | No |
| 42 | XIAP | na |  |  | 16662 | -0.889 | -0.1054 | No |
| 43 | UBE2D3 | na |  |  | 16767 | -0.917 | -0.0877 | No |
| 44 | CTNNB1 | na |  |  | 16890 | -0.952 | -0.0700 | No |
| 45 | FKBP1A | na |  |  | 17150 | -1.031 | -0.0575 | No |
| 46 | BMPR2 | na |  |  | 17365 | -1.107 | -0.0407 | No |
| 47 | RAB31 | na |  |  | 17417 | -1.120 | -0.0151 | No |
| 48 | RHOA | na |  |  | 17463 | -1.139 | 0.0113 | No |
| 49 | TGFBR1 | na |  |  | 18055 | -1.419 | 0.0163 | No |
| 50 | WWTR1 | na |  |  | 18516 | -1.718 | 0.0356 | No |
Table: GSEA details [plain text format]

  

Fig 2: HALLMARK\_TGF\_BETA\_SIGNALING      
 Blue-Pink O' Gram in the Space of the Analyzed GeneSet

  

Fig 3: HALLMARK\_TGF\_BETA\_SIGNALING: Random ES distribution      
 Gene set null distribution of ES for **HALLMARK\_TGF\_BETA\_SIGNALING**
